# Understanding and targeting centrally mediated visceral pain in inflammatory bowel disease

**DOI:** 10.3389/fphar.2014.00027

**Published:** 2014-03-05

**Authors:** Kristen E. Farrell, Robert J. Callister, Simon Keely

**Affiliations:** ^1^School of Biomedical Sciences and Pharmacy, The University of NewcastleCallaghan, NSW, Australia; ^2^Gastrointestinal Research Group, Viruses, Infection/Immunity, Vaccines and Asthma Program, Hunter Medical Research InstituteNew Lambton Heights, NSW, Australia

**Keywords:** inflammatory bowel diseases, colitis, visceral pain, spinal cord, central sensitization

Chronic abdominal pain is a debilitating symptom of inflammatory bowel disease (IBD): a chronic inflammatory condition of the gastrointestinal tract, which includes Crohn's disease (CD) and ulcerative colitis (UC), and is characterized by periods of inflammation and remission. During inflammation, pain is often present due to the activation of afferent nerve endings in the gut by inflammatory mediators (Beyak and Vanner, 2005). Importantly, pain associated with IBD often persists after inflammation has resolved, based on clinical and endoscopic examination, and is often “referred” from the gut to cutaneous or other visceral regions (Minderhoud et al., 2004). Based on what we know about pain processing circuits, this suggests the source of chronic and referred pain lies within the central nervous system (CNS). These clinical observations are supported by work in animal models of visceral inflammation that have provided behavioral, anatomical, molecular, and physiological evidence to implicate spinal circuits in the development of altered pain responses in IBD (Farrell et al., 2014). In spite of this evidence, little is known about the precise mechanisms of chronic pain development and maintenance in IBD. This lack of understanding severely limits current therapeutic approaches for IBD pain management.

## Pain management in IBD

Abdominal pain is a common symptom of IBD, with up to 70% of patients presenting with pain during disease onset, or during periods of relapse (Wagtmans et al., 1998). Pain is an indicator of inflammation, and in the case of IBD, occurs in response to the sensitization of intestinal sensory neurons by inflammatory cytokines. Importantly, inflammation does not appear to be the sole cause of pain in IBD, as 30–50% of patients in clinical remission (i.e., no detectable inflammation in the gut) continue to experience severe abdominal pain (Minderhoud et al., 2004; Farrokhyar et al., 2006; Siegel and MacDermott, 2009). The lack of effective pain management for this patient cohort is problematic in its own right, but is also associated with significantly decreased health-related quality of life scores, increased stress, anxiety, and depression (Farrokhyar et al., 2006; Schirbel et al., 2010). As a consequence, a significant number of IBD patients are chronically treated with narcotics (Edwards et al., 2001; Cross et al., 2005; Makharia, 2011). Unfortunately, long-term narcotic use gives rise to a range of side effects in IBD patients, such as nausea, reduced gastrointestinal motility, and narcotic bowel syndrome; i.e., increased abdominal pain despite escalating narcotic use. These detrimental effects worsen with continued drug use (Grunkemeier et al., 2007), yet the use of narcotics persists, especially for IBD patients during hospitalization and surgery associated with relapse (Lian et al., 2010; Long et al., 2012). These issues are compounded by the lack of alternative pain management therapies, as analgesics such as non-steroidal anti-inflammatory drugs (NSAIDS), including selective COX-2 inhibitors, are linked to disease exacerbation (Bielefeldt et al., 2009; Srinath et al., 2012). Given the lack of options and efficacy issues with current pharmaceutics, it is not surprising that narcotic addiction is a major problem in IBD. Rates of narcotic addiction are over 5% in Crohn's patients and 2.7% overall in IBD patients (Edwards et al., 2001), with the risk increased by 8-fold in those patients with concurrent psychiatric disorders, including depression, anxiety, sexual, emotional and physical abuse and substance abuse (Edwards et al., 2001; Hanson et al., 2009). Thus, there is critical need for new approaches and therapies for IBD pain management. However, before new therapeutics can be designed, the mechanisms responsible for the development and maintenance of chronic abdominal pain in IBD must be understood.

## Sensory transmission in the gastrointestinal tract

The gastrointestinal tract is unique as its function is regulated by both intrinsic and extrinsic components of the autonomic nervous system. Intrinsic innervation is controlled by the enteric nervous system (ENS), which consists of connecting nerve plexuses that run between the muscular layers and the submucosa of the gut wall. The ENS controls gastrointestinal motility, secretion and absorption, which are all essential for gut function, but plays no major role in pain transmission (Blackshaw et al., 2007).

The extrinsic innervation of the gastrointestinal tract includes efferent parasympathetic and sympathetic autonomic pathways that are involved in the modulation of ENS activity. There are also extrinsic sensory afferents that convey information, including (but not exclusive to) noxious (potentially painful) and innocuous sensations, to the spinal cord and brainstem. Brookes et al. have compiled a comprehensive review of extrinsic sensory afferent types that innervate the gut wall (Brookes et al., 2013). Sensory afferents convey signals (including noxious signals that cause pain) from the lower gastrointestinal tract to the CNS via two major nerve trunks, the splanchnic and pelvic nerves (Gebhart, 2000; Blackshaw et al., 2007). These nerves enter the CNS via the spinal cord dorsal horn, where sensory information is subject to extensive modulation via local interneuron networks and descending influences (Melzack and Wall, 1965). Finally, signals from the gut ascend to the cortex via several pathways, including the spinothalamic, spinoreticular and spinomesencephalic tracts, where perception can occur (Craig, 2002; Jones et al., 2006).

## Altered nervous system signaling in IBD-related pain

There is a growing body of evidence from animal studies, suggesting that a dysfunction of the nervous system plays a role in the development of a chronic pain state in IBD (Willis and Westlund, 1997; Gebhart, 1999; Hughes et al., 2009). Most research on the neural mechanisms responsible for pain in IBD has focused on the peripheral nervous system, where strong evidence of hyperexcitability in afferent nerves has been documented. For example, in animal models of chemically induced colitis, hypersensitivity of mechanosensitive sensory afferents following inflammation has been demonstrated, with reduced thresholds for activation and increased frequencies of action potential discharge reported (Hughes et al., 2009; Feng et al., 2012). Importantly, this hypersensitivity is sustained following recovery from inflammation, which may be a contributing factor in the development of chronic pain (Hughes et al., 2009; Feng et al., 2012).

Other work has shown that colonic inflammation can result in increased behavioral responses to mechanical distension and intraluminal administration of capsaicin into the colon, and that this hypersensitivity can be reversed by antagonism of TRPV1 ion channels, implicating TRPV1 expression in the development of peripheral hyperalgesia (Miranda et al., 2007). Taken together, these data indicate that after colonic inflammation, sensory afferents can become hypersensitive to both mechanical and chemical stimulation, and this can be attributed, at least in part, to changes in the expression of ion channels within sensory afferents.

Although the evidence for peripheral contributions to colonic hypersensitivity is strong, the existence of referred pain in IBD patients and in animal models of colitis suggests the CNS, particularly the spinal cord dorsal horn, is implicated in the development of abnormal pain, as referred pain cannot be explained by hypersensitive colon afferents alone (Bernstein et al., 1996). Referred pain is a common sequelae of visceral pain, in which diffuse, poorly localized pain from the viscera “referred” to other areas: most often to the skin (Cervero and Laird, 1999). For IBD, pain is often referred to the mid-lower back, abdomen, and legs (Ritchie, 1973; Ness et al., 1990; Accarino et al., 1995; Bernstein et al., 1996) and, not surprisingly, is a source of great discomfort. It is thought that referred pain occurs when there is overlap between visceral and somatic pathways within the CNS (Traub, 2000). The region of the CNS where this cross over is most likely to occur is within the spinal cord dorsal horn, as the dorsal horn receives inputs from skin, joints and muscle as well as from the viscera (Almeida et al., 2004).

Evidence that the CNS is involved in the processing of inflammation-induced visceral pain has been demonstrated in animal models at behavioral, anatomical, molecular, and physiological levels of analysis (Farrell et al., 2014). Briefly, it has been shown that animals develop referred hypersensitivity of the hind paw and abdomen following colitis (Lamb et al., 2006), and that this sensitivity remains after the resolution of inflammation (Eijkelkamp et al., 2009). Likewise, colitis results in increased dorsal horn expression of markers of neural activation (cFos and pERK) following distension (Traub and Murphy, 2002; Harrington et al., 2012), as well as neuropeptides commonly linked with pain signaling, such as Substance P and CGRP (Sun and Luo, 2004). Finally, the excitability of spinal dorsal horn neurons has been shown to increase following colitis, with extracellular recordings demonstrating a decrease in action potential threshold and increases in spontaneous neural activity (Al-Chaer et al., 1997). We have previously presented a systematic review outlining the evidence for altered CNS activity following gastrointestinal inflammation (Farrell et al., 2014).

Magnetic resonance imaging studies have also demonstrated changes in the structure and functional activation of cortical and subcortical regions in IBD patients compared with healthy controls. Using fMRI, patients with IBD were shown to have differing patterns of cortical activation and deactivation in response to noxious rectal balloon distension (Bernstein et al., 2002). Likewise, CD patients exhibit altered gray matter volumes in cortical (frontal and anterior midcingulate corticies) and subcortical regions that are associated with emotion, cognition, and nociception (Agostini et al., 2013). Therefore, there is evidence for both spinal and supra-spinal alterations as a consequence of chronic pain in IBD.

## Future advances in IBD pain management

The presence of chronic pain in IBD patients represents a major health burden, as it can impact significantly on the quality of life and mental health of long-term sufferers. Current pain management strategies are not optimal: often ineffective and associated with a number of detrimental off-target effects (Edwards et al., 2001; Hanson et al., 2009; Siegel and MacDermott, 2009; Srinath et al., 2012). Recent studies have demonstrated that targeting specific CNS pathways could alleviate visceral pain in animal models. For instance, targeting of central N-methyl-D-aspartate (NMDA) receptors is of particular interest, as there is strong evidence for spinal NMDA receptor-dependent neurotransmission underling the development of central sensitization (Haley et al., 1990; Ren et al., 1992). In models of colonic inflammation, microinjection of the NMDA receptor antagonist DL-2-amino-5-phosphonovaleric acid (APV) into the rostral ventromedial medulla (RVM) was shown to attenuate exaggerated behavioral responses to colon distension. This attenuation was not observed for injections outside the RVM (Coutinho et al., 1998). Since spinal nociceptive transmission is subject to modulation from supraspinal sites such as the RVM, this demonstrates that visceral hyperalgesia can be influenced by NMDA-dependent descending pain modulation. Likewise, cross-organ sensitization between the inflamed colon and the urethra was reversed by intrathecal injection of APV, and by antagonism NR2B: a subunit of the NMDA receptor, using Co-101244 (Peng et al., 2009). Therefore, direct antagonism of spinal NMDA receptors, specifically the NR2B subunit, can attenuate central sensitization caused by inflammation of the colon.

Another potential candidate recently investigated for visceral pain management is the nociceptive ion channel TPVR1. TRPV1 channels are activated by heat (>43°) and capsasin (Christoph et al., 2006) and are expressed in up to 80% of visceral afferents (vs. less than one third in somatic afferents) (Robinson and Gebhart, 2008). This makes these channels an appealing pharmacological target. Silencing of TRPV1 in the CNS using intrathecal injection of TRPV1-specific siRNA 4 days prior to intrarectal capsaicin administration was shown to reduce capsasin-induced spontaneous pain behaviors in rats (Christoph et al., 2006). Importantly, in models of neuropathic pain, TRPV1-specific siRNA analgesia could be maintained for 4–5 days (Christoph et al., 2006). Interestingly, the siRNA used, VsiR1, is directed against a segment of TRPV1 mRNA that is conserved in mouse, rat and human, suggesting this approach may be easily translatable into humans.

Although new CNS targets for managing chronic visceral pain are emerging, the blood brain barrier represents a continual obstacle in the development of new therapeutics. The blood brain barrier presents physical, transport and metabolic barriers that can be modulated and regulated under both normal and pathological states (Abbott et al., 2010). Direct intrathecal injection of therapeutics is the most direct way to bypass these barriers, however, it is by far the least practical solution in a clinical setting. Importantly, recent advances in nanoparticle carrier technologies may allow better targeting of CNS pathways involved in visceral pain through site-directed drug delivery and enhanced penetration of the blood brain barrier (Hua and Wu, 2013).

In summary, visceral pain in chronic inflammatory diseases is poorly understood and current therapeutic strategies are limited. Further research is required to improve our knowledge of the events leading to the development chronic visceral pain. In addition, the discovery of improved CNS targets for pain management along with improved methods for drug delivery, are urgently required for the management IBD patients with chronic pain.
